# Compound K Production from Red Ginseng Extract by β-Glycosidase from *Sulfolobus solfataricus* Supplemented with α-L-Arabinofuranosidase from *Caldicellulosiruptor saccharolyticus*

**DOI:** 10.1371/journal.pone.0145876

**Published:** 2015-12-28

**Authors:** Kyung-Chul Shin, Hye-Yeon Choi, Min-Ju Seo, Deok-Kun Oh

**Affiliations:** Department of Bioscience and Biotechnology, Konkuk University, Seoul 05029, Republic of Korea; Inha University, REPUBLIC OF KOREA

## Abstract

Ginsenoside compound K (C-K) is attracting a lot of interest because of its biological and pharmaceutical activities, including hepatoprotective, antitumor, anti-wrinkling, and anti-skin aging activities. C-K has been used as the principal ingredient in skin care products. For the effective application of ginseng extracts to the manufacture of cosmetics, the PPD-type ginsenosides in ginseng extracts should be converted to C-K by enzymatic conversion. For increased yield of C-K from the protopanaxadiol (PPD)-type ginsenosides in red-ginseng extract (RGE), the α-l-arabinofuranoside-hydrolyzing α-l-arabinofuranosidase from *Caldicellulosiruptor saccharolyticus* (CS-abf) was used along with the β-d-glucopyranoside/α-l-arabinopyranoside-hydrolyzing β-glycosidase from *Sulfolobus solfataricus* (SS-bgly) because SS-bgly showed very low hydrolytic activity on the α-l-arabinofuranoside linkage in ginsenosides. The optimal reaction conditions for C-K production were as follows: pH 6.0, 80°C, 2 U/mL SS-bgly, 3 U/mL CS-abf, and 7.5 g/L PPD-type ginsenosides in RGE. Under these optimized conditions, SS-bgly supplemented with CS-abf produced 4.2 g/L C-K from 7.5 g/L PPD-type ginsenosides in 12 h without other ginsenosides, with a molar yield of 100% and a productivity of 348 mg/L/h. To the best of our knowledge, this is the highest concentration and productivity of C-K from ginseng extract ever published in literature.

## Introduction

Ginseng (*Panax ginseng* C. A. Meyer), one of the most valuable herbs, has been used in traditional medicine in Asian countries for over 2000 years [1]. The active components of ginseng are ginsenosides, which possess diverse biological and pharmaceutical activities, including anti-fatigue [2], anti-allergic [3], anti-oxidant [4], anti-inflammatory [5], anti-cancer [6], and anti-skin aging [7] activities. Ginsenosides are classified into three groups on the basis of the types of the aglycone structures, namely, oleanane, protopanaxadiol (PPD), and protopanaxatriol (PPT). Oleanane, containing oleanolic acid aglycone, exists in only one ginsenoside, that is, Ro. PPD- and PPT-type ginsenosides exist in glycosylated compounds consisting of a non-sugar component with PPD or PPT aglycone and a sugar component with 1−4 glycoside molecules [8]. Glycosylated major ginsenosides (Rb_1_, Rb_2_, Rc, Rd, Rg_1_, and Re) constitute more than 80% of total ginsenosides in wild ginseng [9]. Deglycosylated minor ginsenosides exhibit higher biological activity than major glycosylated ginsenosides because of their smaller size, higher bioavailability, and better permeability across the cell membrane [10]. Commonly, wild ginseng is processed into white ginseng and red ginseng to improve its preservation and efficacy. White ginseng is produced by sun drying, while red ginseng is heated by steaming and then dried. The ginsenoside content in red ginseng differs from that in white ginseng because of the heating process. Red ginseng contains higher content of PPD-type ginsenosides in total ginsenosides than PPT-type ginsenosides, whereas white ginseng contains higher content of PPT-type ginsenosides than PPD-type ginsenosides. The concentration of PPD-type ginsenosides in red ginseng is higher than that in white ginseng. Because red ginseng exerts stronger activities than white ginseng toward anti-skin aging [7], skin damage protection [11], and burn wound healing [12], it has also been used in the production of cosmetics. Red ginseng is fermented using *Lactobacillus* species to enhance the anti-aging potential by [13–15]. Fermented red ginseng increases the content of ginsenosides such as Rg_3_, Rh_1_, F_2_, Rg_2_, and compound K (C-K) [15].

The minor ginsenoside C-K is one of the most biologically and pharmaceutically active PPD-type ginsenosides [16]. C-K has attracted attention in recent years because of hepatoprotective activity [17], inhibition of tumor invasion [18], and induction of tumor cell apoptosis [19]. C-K is also effective against wrinkling and skin damage [20, 21]. Therefore, C-K has been used as the principal ingredient of skin care products. Because C-K is not present in ginseng, the production of C-K from major PPD-type ginsenosides have been intensively tried by biotransformations, including cell conversion [22–24], fermentation [25], and enzymatic conversion [26–28]. Of these methods, enzymatic conversion showed the highest selectivity, yield, and productivity for C-K production. For the industrial production of C-K, ginseng extract should be used as the substrate, instead of purified ginsenosides. However, most studies on C-K production have used purified ginsenosides; only some have used ginseng extracts [29–33]. For the effective application of ginseng extracts to the manufacture of cosmetics, PPD-type ginsenosides in ginseng extracts should be converted to C-K by enzymatic conversion because C-K is an effective agent for anti-wrinkling and anti-skin aging.

In this study, the PPD-type ginsenosides in red ginseng extract (RGE) were completely converted to C-K by the enzymatic reaction involving the β-d-glucopyranoside/α-l-arabinopyranoside-hydrolyzing β-glucosidase from *Sulfolobus solfataricus* (SS-bgly) supplemented with the α-l-arabinofuranoside-hydrolyzing α-l-arabinofuranosidase from *Caldicellulosiruptor saccharolyticus* (CS-abf).

## Materials and Methods

### Materials

The ginsenoside standards Rb_1_, Rb_2_, Rc, Rd, F_2_, compound Mc (C-Mc), and C-K were purchased from BTGin (Daejon, Korea). Red ginseng was purchased from a local ginseng market (Geumsan, Korea). To prepare RGE, 100 g of dry red ginseng powder was added to 1 L of methanol/water mixture (4:1, v/v) and left at 80°C for 1 h. After cooling, the mixture was filtered through a 0.45-μm filter, the filtrate was evaporated to remove methanol, and the residue was dissolved in 1 L of distilled water. The dissolved solution was adsorbed onto a Diaion HP-20 resin, which was further rinsed with distilled water to elute the free sugars. The ginsenosides attached to the resin were successively eluted with methanol, the eluant was evaporated to remove methanol, and the residue was dissolved in the same volume of distilled water as the original loading volume. Sugar-free RGE was used because the Maillard reactions between free sugars and the enzyme occurred at temperatures above 70°C.

### Cloning and gene expression

*S*. *solfataricus* DSM 1617 and *C*. *saccharolyticus* DSM 8903, *Escherichia coli* ER2566, and the plasmids pET-24a and pTrc99A were used as the sources of β-glycosidase and α-l-arabinofuranosidase genes, host cells, and expression vectors, respectively. The genomic DNA from *S*. *solfataricus* and *C*. *saccharolyticus* was extracted using the genomic DNA extraction kit (Qiagen, Hilden, Germany). The genes encoding SS-bgly and CS-abf were amplified from each genomic DNA by polymerase chain reaction (PCR) using *Pfu* DNA polymerase (Solgent, Daejeon, Korea). The sequences of the oligonucleotide primers used for gene cloning were based on the DNA sequences of SS-bgly (GenBank accession number, WP 009992676) and CS-abf (GenBank accession number, YP 001180344). Forward (5'-GCGTCTGCATATGTACTCATTTCCAAATAGC-3') and reverse (5'-GCGAATGGATCCTTAGTGCCTTAATGGCTTTAC-3') primers were designed to introduce the *Nde*Ι and *BamH*Ι restriction sites (underlines), respectively, for SS-bgly gene insertion, and forward (5'-TTTGGATCCATGAAAAAAGCAAAAGTCATCTA-3') and reverse (5'-TTTCTGCAGTTAATTTTCTCTCTTCTTCAATCTG-3') primers were designed to introduce the *BamH*Ι and *Pst*Ι restriction sites (underlined), respectively, for CS-abf gene insertion. Each amplified DNA fragment obtained by PCR was purified and digested with the corresponding restriction endonuclease [34, 35]. The digested DNA fragments of SS-bgly and CS-abf were extracted using a gel extraction kit (Qiagen, Hilden, Germany), and then inserted into the pET-24a vector and pTrc99A vectors, respectively, and digested with the same restriction enzymes. *E*. *coli* ER2566 strain was transformed with the ligation mixture and plated on Luria-Bertani (LB) agar containing 50 μg/mL of ampicillin. Ampicillin resistant colony was selected, and plasmid DNA from the transformant was isolated using a plasmid purification kit (Promega, Madison, WI). DNA sequencing was performed at the Macrogen facility (Seoul, Korea). The expression of SS-bgly and CS-abf genes was evaluated by both sodium dodecyl sulfate-polyacrylamide gel electrophoresis (SDS-PAGE) and enzyme activity assay.

### Culture conditions

*S*. *solfataricus* and *C*. *saccharolyticus* were grown at 70°C under anaerobic conditions in the presence of 100% N_2_ gas for 5 days on *Sulfolobus* medium (DSM Media Formulation No. 88) and *Caldicellulosiruptor* medium (DSM Media Formulation No. 640), respectively. The recombinant *E*. *coli* was cultivated with shaking at 200 rpm in a 2-L flask containing 500 mL of LB medium at 37°C with 20 μg/mL of kanamycin. When the optical density of the bacteria at 600 nm reached 0.8, isopropyl-β-d-thiogalactopyranoside (IPTG) was added to a final concentration of 0.1 mM to induce enzyme expression, and the culture was incubated at 16°C with shaking at 150 rpm for 16 h.

### Enzyme purification

The induced *E*. *coli* cells were harvested from the culture broth by centrifugation at 6,000*g* for 30 min at 4°C, washed twice with 0.85% NaCl, and resuspended in 50 mM citrate/phosphate buffer (pH 6.0) containing 0.1 mM phenylmethylsulfonyl fluoride as the protease inhibitor. The resuspended cells were disrupted by sonication using Sonic Dismembrator (Model 100, Fisher Scientific, Pittsburgh, PA, USA) on ice for 10 min. The unbroken cells and cell debris were removed by centrifugation at 13,000×*g* for 20 min at 4°C, and the supernatant obtained was used as the crude extract. The crude extract was subsequently heated at 75°C for 10 min, and the suspension was centrifuged at 13,000×*g* for 20 min to remove insoluble denatured proteins. The supernatant obtained was used as the soluble enzyme.

### Enzyme reactions

One unit (U) of SS-bgly or CS-abf activity was defined as the amount of enzyme required to liberate 1 nmol of C-K or ginsenoside Rd as a product from ginsenoside Rd or Rc as a substrate, respectively. Unless otherwise stated, the reactions were performed at 80°C in 50 mM citrate/phosphate buffer (pH 6.0) containing 1.25 g/L PPD-type ginsenosides in RGE for 2 h.

### Optimization of the supplementation ratio of SS-bgly and CS-abf

The effect of the supplementation ratio of SS-bgly and CS-abf on the conversion of ginsenoside Rc to C-K was investigated by the enzymatic reactions of SS-bgly supplemented with CS-abf at the ratios of 0:2 to 2:0 U/mL, at a total concentration of 2 U/mL. The reactions were performed at 80°C in 50 mM citrate/phosphate buffer (pH 6.0) containing 0.4 g/L ginsenoside Rc and two enzymes, at a total concentration of 2 U/mL, for 1 h. The time-course reactions for the conversion of PPD-type ginsenosides in RGE to C-K were performed at 80°C in 50 mM citrate/phosphate buffer (pH 6.0) containing 1.25 g/L PPD-type ginsenosides in RGE, 0.4 U/mL SS-bgly, and 1.6 U/mL CS-abf in 20 h. The effect of the supplementation ratio of CS-abf to SS-bgly on the production of C-Mc from PPD-type ginsenosides in RGE was conducted by supplementing CS-abf, ranging from 0.0 to 1.6 U/mL, with 0.4 U/mL SS-bgly. The reactions were performed with 1.25 g/L PPD-type ginsenosides in RGE for 20 h.

### Effects of pH and temperature on C-K production

The effects of pH and temperature on the production of C-K from PPD-type ginsenosides in RGE were investigated by varying the pH from 4.5 to 7.5, and the temperature from 65 to 95°C, respectively. The reactions were performed in 50 mM citrate/phosphate buffer containing 0.4 U/mL SS-bgly, 0.6 U/mL CS-abf, and 1.25 g/L PPD-type ginsenosides in RGE for 2 h. The effect of temperature on the stability of SS-bgly supplemented with CS-abf was monitored as a function of incubation time by maintaining the solution of the enzymes at five different temperatures (70, 75, 80, 85, and 90°C) in 50 mM citrate/phosphate buffer (pH 6.0). Samples were withdrawn at time intervals and assayed.

### Effects of the concentrations of enzymes and substrate on C-K production

The optimal concentration of the two enzymes required for C-K production was determined by varying the concentrations of SS-bgly and CS-abf at the ratio of 1:1.5, ranging from 0.4 and 0.6 U/mL to 4 and 6 U/mL at a constant substrate concentration of 7.5 g/L PPD-type ginsenosides in RGE. To determine the optimal substrate concentration, the concentration of PPD-type ginsenosides was varied from 1.25 to 10 g/L at constant enzyme concentrations of 2 U/mL SS-bgly and 3 U/mL CS-abf. The reactions were performed in 50 mM citrate/phosphate buffer (pH 6.0) at 80°C for 2 h.

### Production of C-K from PPD-type ginsenosides in RGE

The time-course reactions for the conversion of PPD-type ginsenosides in RGE to C-K were performed at 80°C in 50 mM citrate/phosphate buffer (pH 6.0) containing 7.5 g/L PPD-type ginsenosides in 17.4% (w/v) RGE and 2 U/mL SS-bgly or 2 U/mL SS-bgly supplemented with 3 U/mL CS-abf for 12 h.

### HPLC analysis

A reaction solution containing digoxin as an internal standard was extracted with an equal volume of *n*-butanol. The *n*-butanol fraction was then evaporated until dry, and methanol was added. Ginsenosides were assayed using an HPLC system (1100, Agilent, Santa Clara, CA, USA) equipped with a UV detector at a detection wavelength of 203 nm and a C18 column (YMC, Kyoto, Japan). The column was eluted at 37°C with a linear gradient of acetonitrile/water from 20:80 to 80:20 (v/v) for 80 min at a flow rate of 1 mL/min.

### TLC analysis

Three microliters of the ginsenoside standards and the reaction products of ginsenoside Rc were spotted onto a TLC plate (silica gel 60 F254, Merck, Darmstadt, Germany). The compounds on the plate were developed using a solvent mixture of chloroform, methanol, and water (40:20:10, v/v/v). The spots on the plate were detected by spraying 10% sulfuric acid, followed by incubation at 110°C in a dry oven for 5 min.

## Results and Discussion

### Conversion of major PPD-type ginsenosides to C-K by SS-bgly supplemented with CS-abf

**Fig 1:**
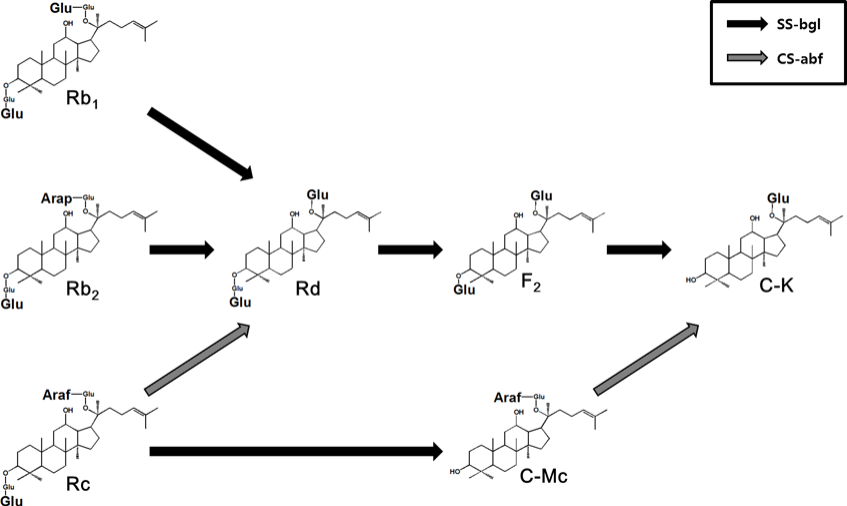
Hydrolytic pathway from ginsenosides Rb_1_, Rb_2_, and Rc to C-K via C-Mc, ginsenoside Rd, and F_2_ by SS-bgly supplemented with CS-abf.

SS-bgly is a useful enzyme for C-K production because the enzyme simultaneously hydrolyzes the outer β-d-glucopyranoside or α-l-arabinopyranoside linked to C-20, and the inner and outer β-d-glucopyranosides linked to C-3 in PPD-type ginsenosides [32]. However, the enzyme exhibits very low hydrolytic activity for α-l-arabinofuranoside in PPD-type ginsenosides, indicating that SS-bgly does not convert ginsenosides Rc and C-Mc to Rd and C-K, respectively. Thus, CS-abf, which showed hydrolytic activity for α-l-arabinofuranoside [36], was supplemented to SS-bgly for the complete conversion of all PPD-type ginsenosides to C-K (Fig 1).

SS-bgly and CS-abf were purified as soluble proteins by heat treatment of crude enzymes extracted from harvested cells. The conversion of ginsenoside Rc to C-K by the enzymatic reaction of SS-bgly supplemented with CS-abf was analyzed and confirmed by TLC using the ginsenoside standards Rc, Rd, F_2_, C-Mc, and C-K (S1 Fig). Ginsenosides Rd, F_2_ (trace), C-Mc, and C-K were obtained from ginsenoside Rc by SS-bgly supplemented with CS-abf, whereas only C-Mc was obtained by SS-bgly alone.

These results indicate that the supplementation of CS-abf to SS-bgly is an effective method for C-K production by introducing the α-l-arabinofuranoside-hydrolyzing activity, which caused the conversion of Rc and C-Mc to Rd and C-K, respectively.

### Optimization of the supplementation ratio of SS-bgly and CS-abf for C-K production

**Fig 2:**
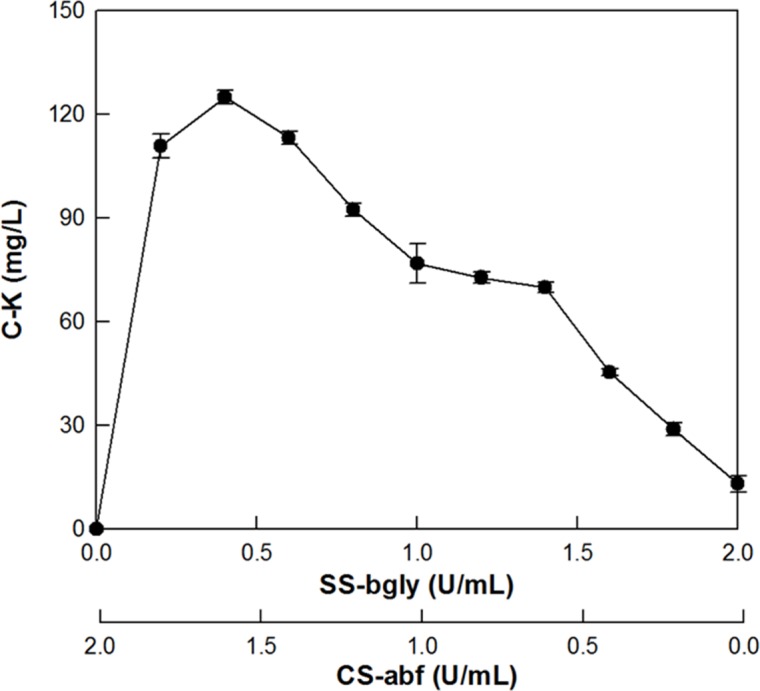
Effect of supplementation of CS-abf to SS-bgly on the conversion of ginsenoside Rc to C-K. Data represent the means of three experiments and error bars represent standard deviation.

**Fig 3:**
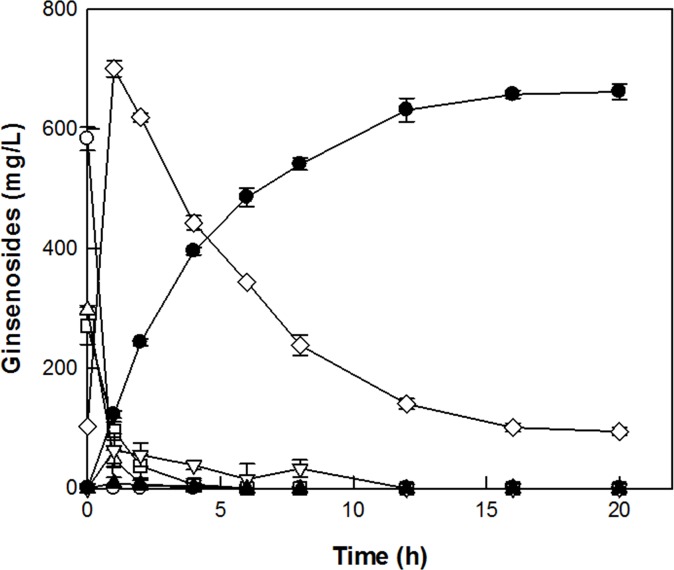
Production of C-K from PPD-type ginsenosides in RGE by SS-bgly supplemented with CS-abf. Ginsenosides Rb_1_ (open circle), Rb_2_ (open square), Rc (open triangle), and Rd (open diamond) were converted to C-K (filled circle) via F_2_ (open inverted triangle) and C-Mc (filled triangle). Data represent the means of three experiments and error bars represent standard deviation.

**Fig 4:**
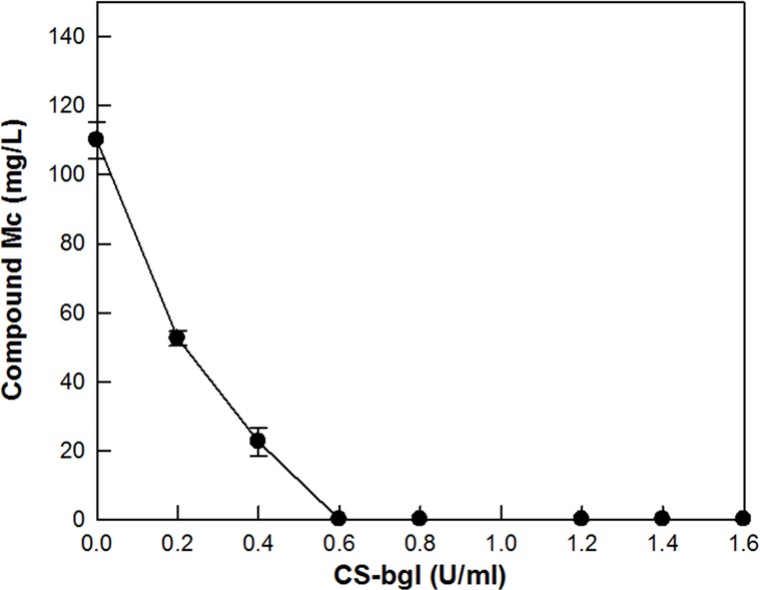
Effect of supplementation of CS-abf to SS-bgly on the concentration of C-Mc in RGE. Data represent the means of three experiments and error bars represent standard deviation.

The production of C-K from ginsenoside Rc was investigated by supplementing CS-abf to SS-bgly at the ratios ranging from 0:2 to 2:0 U/mL, to make up a total concentration of 2 U/mL. The maximum production of C-K was observed at 0.4 U/mL SS-bgly and 1.6 U/mL CS-abf with a unit ratio of 1:4 (Fig 2). This ratio was used in the conversion of PPD-type ginsenosides in RGE to C-K (Fig 3). In the conversion, ginsenoside Rc and Mc disappeared after 2 h, whereas ginsenoside Rd remained at a constant concentration of approximately 100 mg/L even after 16 h. These results suggest that the concentration of CS-abf added to the reaction solution for C-K production was excess compared to that of SS-bgly. To optimize the supplementation ratio of SS-bgly and CS-abf for the production of C-K from PPD-type ginsenosides in RGE, the residual concentration of C-Mc was determined after 20 h by increasing the concentration of CS-abf from 0.0 to 1.6 U/mL, and maintaining a fixed concentration of SS-bgly at 0.4 U/mL. The residual concentration of C-Mc decreased with increasing the concentration of CS-abf, at concentrations below 0.6 U/mL CS-abf. However, C-Mc was not detected at concentrations above 0.6 U/mL CS-abf (Fig 4). Therefore, the optimal unit ratio of SS-bgly and CS-abf was 0.4 U/mL: 0.6 U/mL (1:1.5) for the production of C-K from PPD-type ginsenosides in RGE, respectively. This optimal unit ratio was used in the subsequent reactions.

### Effects of pH and temperature on C-K production by SS-bgly supplemented with CS-abf

**Fig 5:**
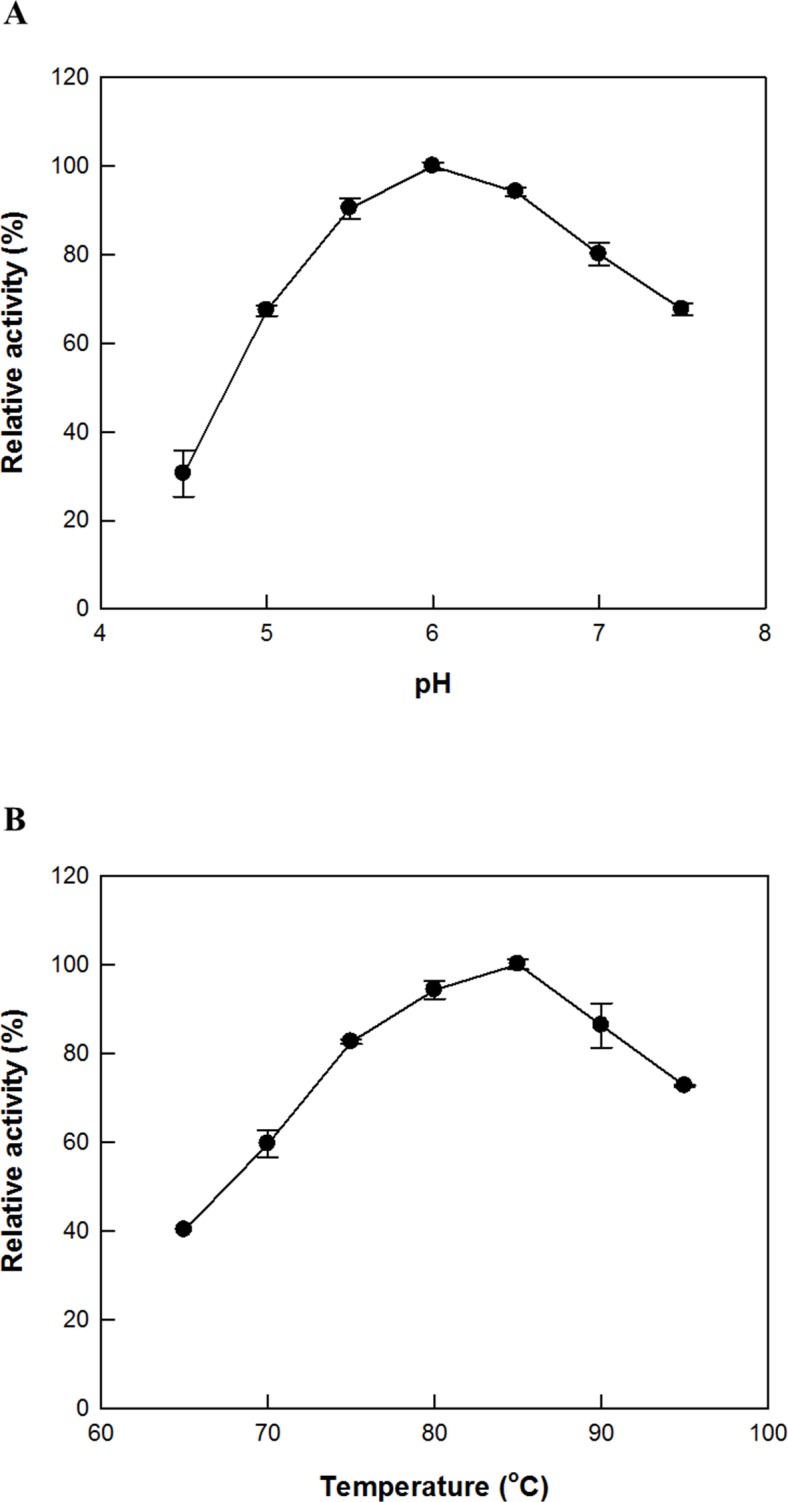
Effects of (A) pH and (B) temperature on the production of C-K from PPD-type ginsenosides in RGE by SS-bgly supplemented with CS-abf. Data represent the means of three experiments and error bars represent standard deviation.

The maximum production of C-K by the enzymatic reaction of SS-bgly supplemented with CS-abf at the optimal unit ratio was observed at pH 6.0 and 85°C (Fig 5), and the optimal pH and temperature of only SS-bgly were also 6.0 and 85°C, respectively. The thermal inactivation of SS-bgly supplemented with CS-abf at the optimal unit ratio for the production of C-K from PPD-type ginsenosides in RGE followed first-order kinetics, with half-lives of 346, 76, 25, 15, and 1.2 h at 70, 75, 80, 85, and 90°C (S2 Fig). The half-lives of SS-bgly at 70, 75, 80, and 85°C were 700, 100, 66, and 30 h [32]; and those of CS-abf were 130, 41, 2.5, and 0.12 h [36]. Thus, the thermostability of SS-bgly supplemented with CS-abf was greater than that of CS-abf alone, but less than that of SS-bgly alone. Because of the instability of SS-bgly supplemented with CS-abf above 85°C, the reaction temperature for C-K production by SS-bgly supplemented with CS-abf was determined to be 80°C.

### Effects of the concentrations of enzymes and substrate on C-K production by SS-bgly supplemented with CS-abf

**Fig 6:**
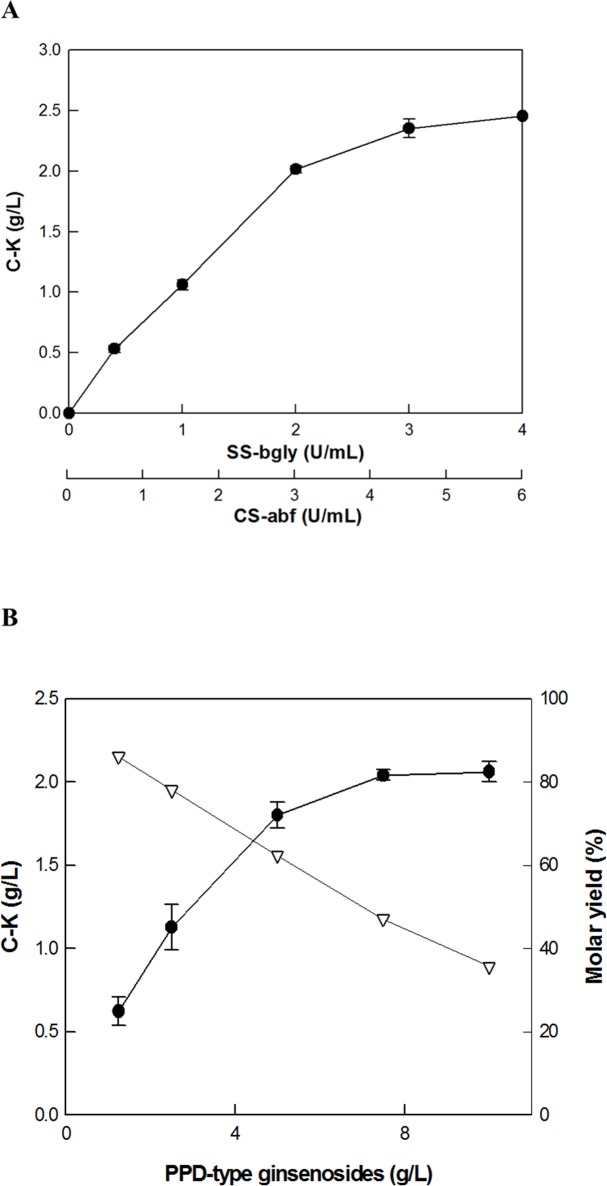
Effect of the concentration of (A) SS-bgly supplemented with CS-abf and (B) PPD-type ginsenosides in RGE. Filled circle and open inverted triangle represent concentration of C-K and conversion yield, respectively. Data represent the means of three experiments and error bars represent standard deviation.

The effect of the concentration of SS-bgly supplemented with CS-abf on C-K production was investigated with 7.5 g/L PPD-type ginsenosides in RGE as a substrate by varying the concentrations of SS-bgly and CS-abf at the optimal unit ratio of 1:1.5, ranging from 0.4 and 0.6 U/mL to 4 and 6 U/mL, respectively (Fig 6A). C-K production increased with increasing enzyme concentrations, however, C-K production per enzyme concentration decreased at concentrations above 2 U/mL SS-bgly and 3 U/mL CS-abf. Thus, the optimal concentration of the enzymes required for the production of C-K from PPD-type ginsenosides in RGE was 2 U/mL SS-bgly and 3 U/mL CS-abf.

To investigate the effect of substrate concentration on C-K production, the concentration of PPD-type ginsenosides in RGE was varied from 1.25 to 10 g/L. The conversion yield decreased with increasing the substrate concentration (Fig 6B). However, the maximal production of C-K was exhibited above 7.5 g/L PPD-type ginsenosides. Thus, the optimal concentration of substrate was 7.5 g/L PPD-type ginsenosides in RGE.

### C-K production from PPD-type ginsenosides in RGE by SS-bgly supplemented with CS-abf under optimum conditions

**Fig 7:**
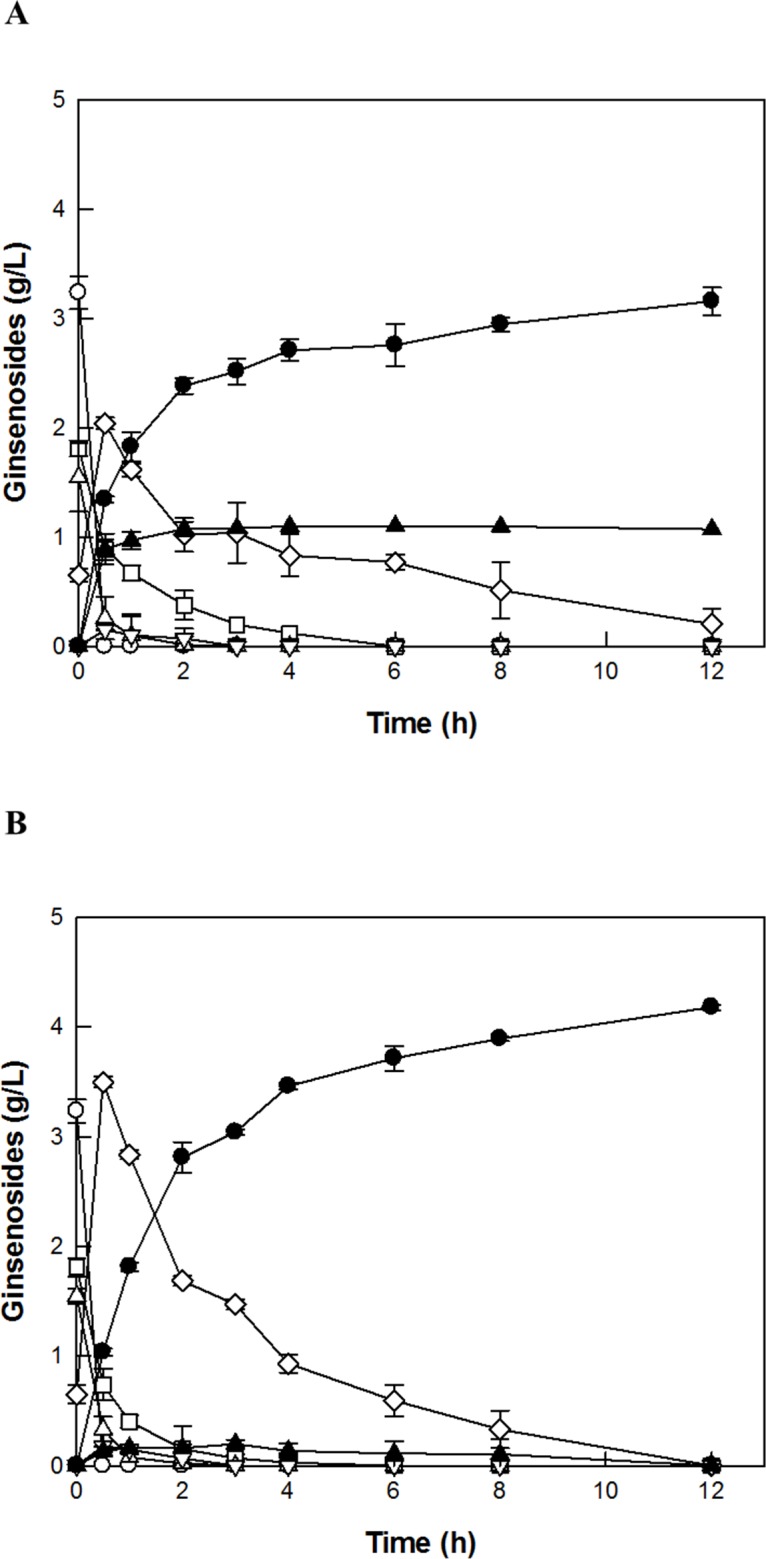
C-K production from PPD-type ginsenosides in RGE. C-K (filled circle) was produced from ginsenosides Rb_1_ (open circle), Rb_2_ (open square), Rc (open triangle), and Rd (open diamond) in RGE via F_2_ (open inverted triangle) and C-Mc (filled triangle) by (A) SS-bgly. (B) SS-bgly supplemented with CS-abf under the optimized unit ratio of the two enzymes. Data represent the means of three experiments and error bars represent standard deviation.

The optimal reaction conditions for the production of C-K from PPD-type ginsenosides in RGE by SS-bgly supplemented with CS-abf were pH 6.0, 80°C, 2 U/mL SS-bgly, 3 U/mL CS-abf, and 7.5 g/L PPD-type ginsenosides in RGE. Under these conditions, 2 U/mL SS-bgly without supplementation of CS-abf produced 3.1 g/L C-K from 7.5 g/L PPD-type ginsenosides in RGE along with other ginsenosides, 1.1 g/L C-Mc and 0.3 g/L Rd, after 12 h, with a molar yield of 75% and a productivity of 263 mg/L/h for the production of C-K from PPD-type ginsenosides (Fig 7A). To convert the remaining other ginsenosides to C-K, 3 U/mL CS-abf was supplemented to 2 U/mL SS-bgly. Under the optimized conditions, SS-bgly supplemented with CS-abf converted 7.5 g/L PPD-type ginsenosides in RGE to 4.2 g/L C-K in 12 h, with a molar yield of 100% and a productivity of 348 mg/L/h (Fig 7B). The molar yield and productivity of C-K by SS-bgly supplemented with CS-abf were 25% and 1.3-fold higher, respectively, than those by SS-bgly alone. No C-K was formed when the reaction was run under the same experimental conditions without enzymes.

The production of C-K from PPD-type ginsenosides in ginseng extracts by enzymes and cells is presented in Table 1. C-K was also synthesized from glucose by metabolically engineered yeast, however, the produced concentration of C-K was low (1.4 mg/L) [37]. β-Glycosidase from *Sulfolobus acidocaldarius* (SA-bgly) produced 2.0 g/L C-K from 5 g/L PPD-type ginsenosides in ginseng root extract (GRE) in 22 h, with a molar yield of 69% and a productivity of 91 mg/L/h [38], which were 6% and 2.9-fold lower than those in RGE by SS-bgly. α-l-Arabinopyranoside linked to ginsenoside Rb_2_ could be hydrolyzed by SS-bgly but not by SA-bgly. The content of Rc among the PPD-type ginsenosides such as Rb_1_, Rb_2_, Rc, and Rd in RGE (21.3%) was lower than that in GRE (26.9%), and the concentration of the PPD-type ginsenosides in 10% (w/v) RGE was 1.5-fold higher (Table 2), indicating that RGE is a better substrate for C-K production. Thus, SS-bgly using RGE as the substrate exhibited higher conversion yield and productivity of C-K than SA-bgly using GRE. Diverse glycosidases from *Fusobacterium* sp. [39], *Microbacterium esteraromaticum* [28], *S*. *acidocaldarius* [29], *Aspergillus niger* [40], *Aspergillus* sp. [41], *Esteya vermicola* [42], and *Terrabacter ginsenosidimutans* [43] converted reagent-grade major ginsenosides Rb_1_, Rb_2_, Rc, and Rd to C-K. However, the quantitative production of C-K from Rc was conducted by only the combined use of three-enzymes [38]. The three-enzyme system of SA-bgly, and α-l-arabinofuranosidase and β-galactosidase from *C*. *saccharolyticus* produced 2.9 g/L C-K from 5 g/L PPD-type ginsenosides in GRE in 20 h, with a molar yield of 100% and a productivity of 144 mg/L/h [38]. To the best of our knowledge, this was the highest previously reported conversion yield and productivity of C-K from ginseng extract. The concentration and productivity of C-K as obtained by the two-enzyme system of SS-bgly and CS-abf using RGE, which contained the higher content of PPD-type ginsenosides than GRE, were 1.4- and 2.4-fold higher than those obtained by the three-enzyme system using GRE, respectively. The supplementation of CS-abf to SS-bgly resulted in the complete conversion of all PPD-type ginsenosides in RGE to C-K with the highest productivity ever reported via two transformation pathways: Rb_1_, Rb_2_, and Rc → Rd → F_2_ → C-K and Rc → C-Mc → C-K (Fig 1). The conversion of ginsenosides Rb_1_, Rb_2_, Rc, and Rd in RGE to C-K via F_2_, and C-Mc was analyzed and confirmed by HPLC with the ginsenoside standards (S3 Fig).

**Table 1:** Production of C-K from PPD-type ginsenosides in ginseng extracts.

Microorganism	Biocatalyst	Extract	C-K (g/L)	Molar yield (%)	Productivity (mg L^−1^ h^−1^)	Reference
*Aspergillus niger*	Pectinase	Rootlet ginseng	2.0	NC	54	[44]
*Aspergillus niger*	Cytolase PCL 5	White ginseng extract	2.1	NC	27	[45]
*Sulfolobus acidocaldarius*,	β-Glycosidase	Ginseng root extract	2.0	69	91	[38]
*Sulfolobus acidocaldarius* and *Caldicellulosiruptor saccharolyticus*	β-Glycosidase, α-l-arabinofuranosidase, and β-galactosidase	Ginseng root extract	2.9	100	144	[38]
*Saccharomyces cerevisiae*	Cells	Red ginseng extract	0.3	NC	3	[46]
*Paecilomyces bainier* sp.	Cells	Notoginseng extract	1.3	83	9	[47]
*Ganoderma lucidum*	Cells	American ginseng root extract	0.7	2	1	[48]
*Sulfolobus solfataricus*	β-Glycosidase	Red ginseng extract	3.1	75	263	This study
*Sulfolobus solfataricus* and *Caldicellulosiruptor saccharolyticus*	β-Glycosidase and α-l-arabinofuranosidase	Red ginseng extract	4.2	100	348	This study

NC, not calculated.

**Table 2:** Contents of PPD- and PPT-type ginsenosides in 10% (w/v) RGE and GRE.

Ginsenoside	RGE				GRE	
	Concentration (g/L)	Content^*a*^ (%, w/w)	Content^*b*^ (%, w/w)	Concentration (g/L)	Content^*a*^ (%, w/w)	Content^*b*^ (%, w/w)
PPD-type						
Rb_1_	1.92	27.4	44.5	1.39	21.0	46.8
Rb_2_	1.08	15.4	25.1	0.49	7.4	16.5
Rc	0.92	13.1	21.3	0.80	12.1	26.9
Rd	0.39	5.6	9.1	0.29	4.4	9.8
Subtotal	4.31	61.6	100.0	2.97	44.9	100.0
PPT-type						
Rg_1_	0.61	8.7		1.83	27.6	
Rh_1_	0.12	1.7		0.06	0.9	
Re	1.78	25.4		1.66	25.1	
Rg_2_	0.18	2.6		0.10	1.5	
Subtotal	2.69	38.4		3.65	55.1	
Total	7.00	100.0		6.62	100.0	

^*a*^Contents of total ginsenosides.

^*b*^Contents of each type of ginsenosides.

## Conclusions

SS-bgly shows hydrolytic activity on the β-d-glucopyranoside and α-l-arabinopyranoside linkage in ginsenosides without the α-l-arabinofuranoside linkage. To compensate this defect, SS-bgly was supplemented with α-l-arabinofuranoside-hydrolyzing CS-abf. This combination completely converted PPD-type ginsenosides in RGE to C-K. To the best of our knowledge, this is the highest concentration and productivity of C-K from ginseng extract ever published in literature. Our results would contribute toward improving the industrial production of C-K by biotransformation.

## Supporting Information

S1 Fig TLC analysis of the reaction products of ginsenoside Rc. Ginsenoside Rc was converted to the reaction products by only SS-bgly and SS-bgly supplemented with CS-abf. *Lane S*, ginsenoside standards; *Lane 1*, reaction products of ginsenoside Rc by SS-bgl alone; *Lane 2*, reaction products of ginsenoside Rc by SS-bgly supplemented with CS-abf.
(TIF)

S2 Fig Thermal inactivation of the activity of SS-bgly supplemented with CS-abf. The enzymes were incubated at 70°C (open square), 75°C (filled square), 80°C (open circle), 85°C (filled circle), and 90°C (filled triangle) for varying time periods. Data represent the means of three experiments and error bars represent standard deviation.
(TIF)

S3 Fig HPLC profiles of the C-K produced from PPD-type ginsenosides in RGE by SS-bgly supplemented with CS-abf. HPLC analysis of C-K (27.9 min) produced from ginsenosides Rb_1_ (9.6 min), Rb_2_ (11.9 min), Rc (11.4 min), and Rd (12.8 min) present in RGE via F_2_ (18.1 min) and compound Mc (23.4 min) by SS-bgly supplemented with CS-abf at 0, 2, and 12 h.
(TIF)
