# Supplementary figures and images for: Compound K Production from Red Ginseng Extract by β-Glycosidase from Sulfolobus solfataricus Supplemented with α-L-Arabinofuranosidase from Caldicellulosiruptor saccharolyticus
Source: PLoS One. 2015 Dec 28;10(12):e0145876. doi: 10.1371/journal.pone.0145876 (PMC4692446; doi:10.1371/journal.pone.0145876)

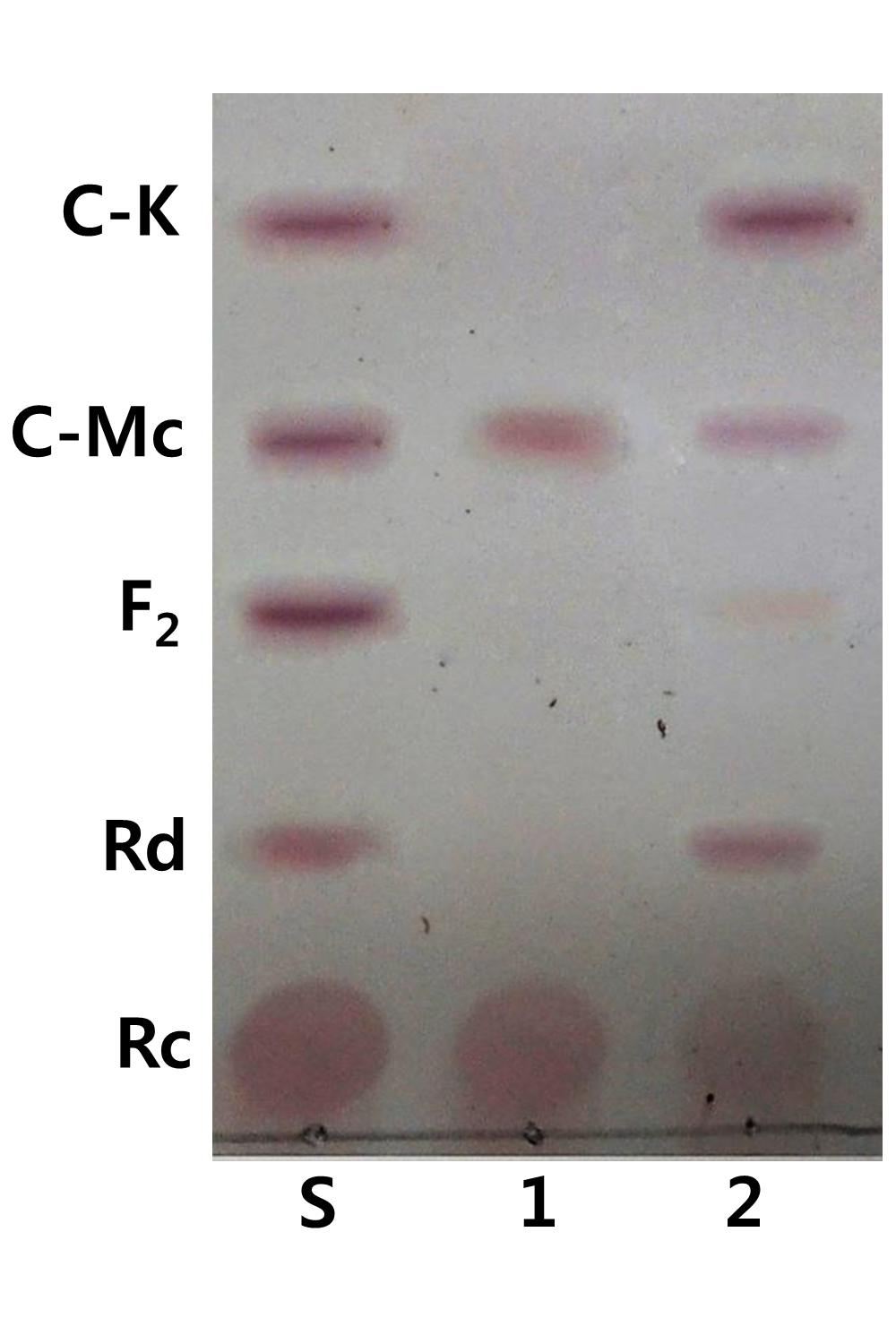

Supplement: S1 Fig — Ginsenoside Rc was converted to the reaction products by only SS-bgly and SS-bgly supplemented with CS-abf. Lane S, ginsenoside standards; Lane 1, reaction products of ginsenoside Rc by SS-bgl alone; Lane 2, reaction products of ginsenoside Rc by SS-bgly supplemented with CS-abf. (TIF) [file pone.0145876.s001.tif]

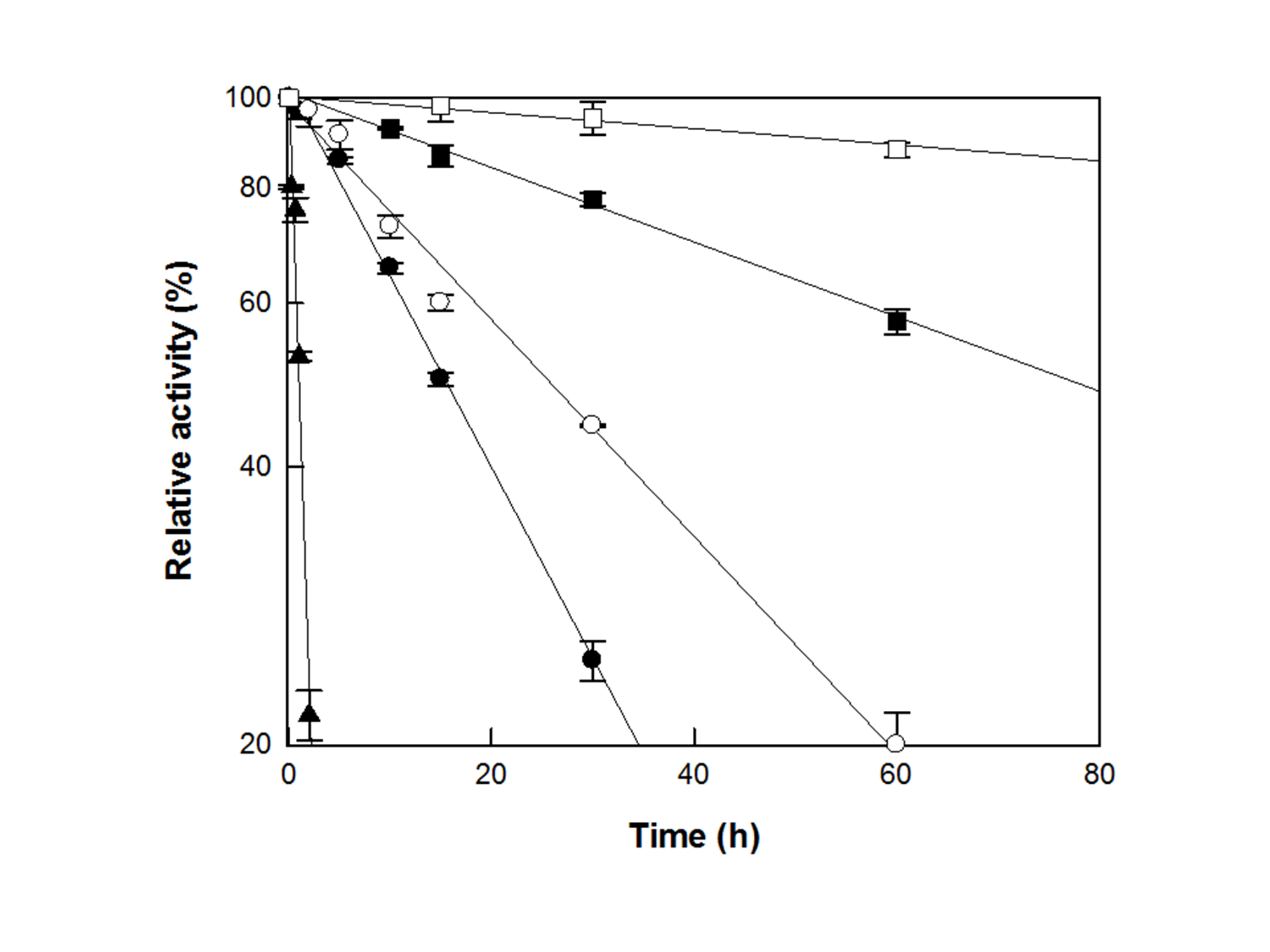

Supplement: S2 Fig — The enzymes were incubated at 70°C (open square), 75°C (filled square), 80°C (open circle), 85°C (filled circle), and 90°C (filled triangle) for varying time periods. Data represent the means of three experiments and error bars represent standard deviation. (TIF) [file pone.0145876.s002.tif]

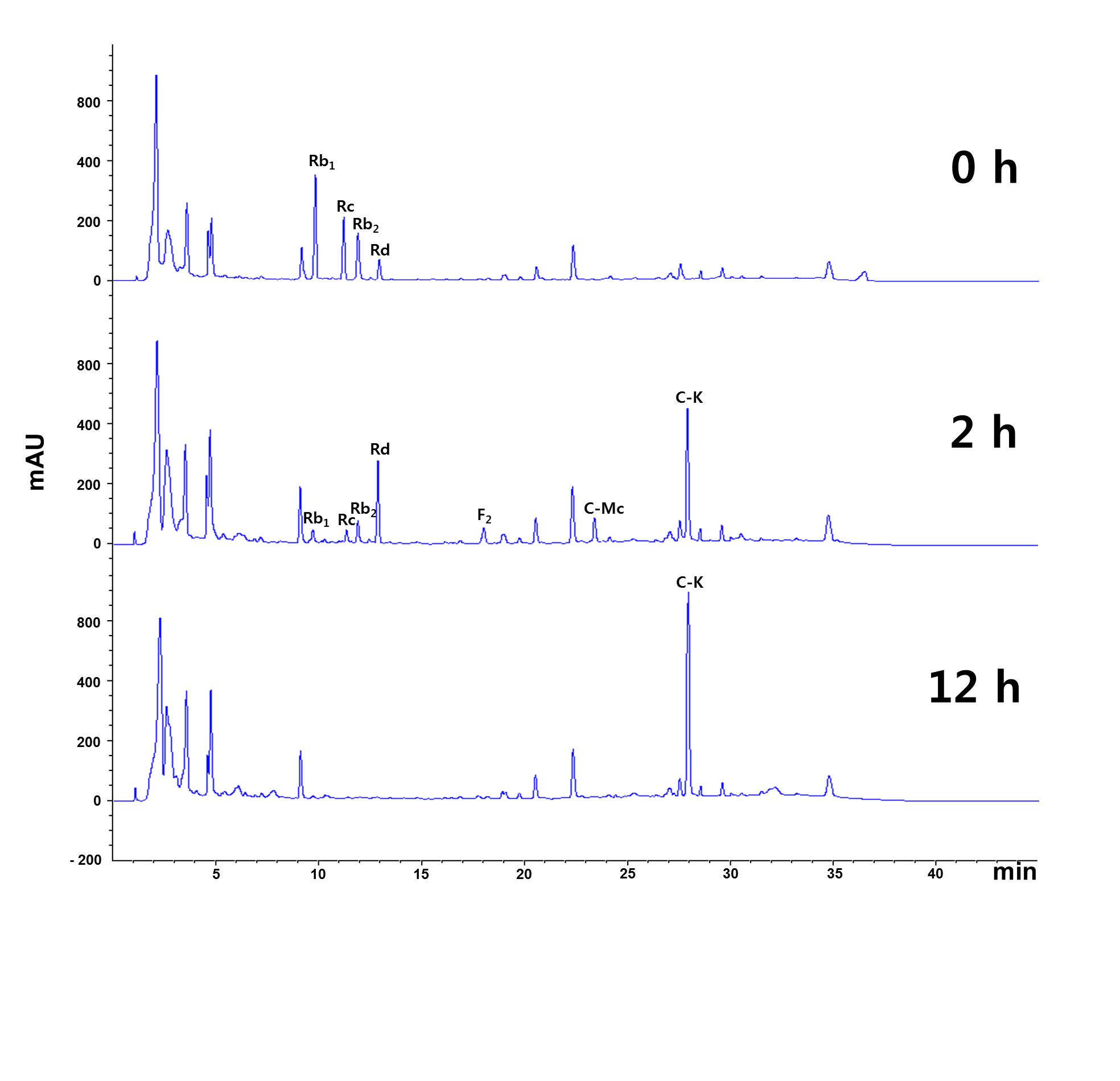

Supplement: S3 Fig — HPLC analysis of C-K (27.9 min) produced from ginsenosides Rb1 (9.6 min), Rb2 (11.9 min), Rc (11.4 min), and Rd (12.8 min) present in RGE via F2 (18.1 min) and compound Mc (23.4 min) by SS-bgly supplemented with CS-abf at 0, 2, and 12 h. (TIF) [file pone.0145876.s003.tif]
